# On the Moral and Physical Character (Cerebral Development) of Burk

**Published:** 1829-07-01

**Authors:** 


### On the Moral and Physical Character (Cerebral Development) of Burk.

In the last number of the Phrenological Journal, there is a very able and interesting article on the above subject. We do not pretend to any intimate knowledge of phrenology—not indeed having sufficient leisure for the study; but we conceive ourselves competent to form an opinion as to the truth or falsehood of the main principle on which it rests, as a science. We believe that the controversy between the phrenologists and antiphrenologists might be narrowed at once to the discussion of a single question. It is now, we should conceive, generally admitted, that our passions, propensities, dispositions are born with us, however some of them may become more developed than others as we grow up, and as chance or tuition may call them into activity. Now, the question is this:—in what part of man are these passions or propensities located? Are they connected with material organs — or were they implanted in the mind, the soul, the divinæ particula auræ, when it was first infused into the primary rudiment of our material fabric? It is said that the doctrine which assigns material organs or parts to the passions or propensities, is an immoral one; for why should we be responsible for the operation of these propensities, when their seats or causes form a part of our bodies? But we apprehend that the same argument will turn very strongly indeed against the opposite party and doctrine. The existence of good and evil propensities is acknowledged; and if they are implanted or located in the immortal soul by the hand of our Creator, we are surely as little accountable for their operations as if they were connected or seated in our corporeal structure. At one time it was treason to say that insanity was a *corporeal* malady; and yet it is now almost unanimously considered as dependent on derangement of body. If, then, we see inflammation, or other disease of the brain, alter, confuse, augment, or diminish various passions, propensities, and intellectual faculties, is it not reasonable to suppose that these passions, &c. are connected with, and manifested through certain material structures, whether we are able to ascertain these localisations or not?

We imagine that our morality or immorality — our responsibility or irresponsibility, is not affected, after all, by the location of our passions in either body or mind. Good and bad passions —good and bad organs, come into the world with us, and we should suppose that our responsibility will hinge on our endeavours to make the good preponderate over the evil portions of our nature. This, we believe, is the doctrine of phrenology as well as of religion. Even the poet has laid it down in two expressive lines:—

"Two principles in human nature reign,
"Passion to urge, and reason to restrain."

Let us hear what the learned and pious Dr. Blair says:—

"The seeds of various qualities, good and bad, lie in all our hearts; but until proper occasions ripen and bring them forward, they lie there inactive and dead. They are covered up and concealed within the recesses of our nature; or, if they spring up at all, it is under such an appearance as is frequently mistaken, even by ourselves. Pride, for instance, in certain situations, has no opportunity of displaying itself, but as magnanimity, or sense of honour. Avarice appears as necessary and laudable economy. What in one station of life would discover itself to be cowardice and baseness of mind, passes in another for prudent circumspection. What in the fulness of power would prove to be cruelty and oppression, is reputed, in a subordinate rank, no more than the exercise of proper discipline. For a while the man is known neither by the world nor by himself to be what he truly is. But bring him into a new situation of life, which accords with his predominant dispositions, which strikes on certain latent qualities of his soul, and awakens them into action; and as the leaves of a flower gradually unfold to the sun, so shall all his true character open full to view.

"This may in one light be accounted not so much an alteration of character produced by a change of circumstances as a discovery brought forth of the *real character*, which formerly lay concealed. Yet, at the same time, it is true that the man himself undergoes a change. For the opportunity being given for certain dispositions, which had been dormant, to exert themselves without restraint, they of course gather strength. By means of the ascendancy which they gain, other parts of the temper are borne down, and thus an alteration is made in the whole structure and system of the soul." 12*th Sermon.*

Now what are these "seeds of various qualities, good and bad" which "lie in all our hearts?" Do they lie in the immortal soul—or in the mortal body? And what can Dr. Blair mean by "an alteration made in the whole *structure* and system of the soul?" He appears to shadow out the modern doctrine of phrenology—for we cannot imagine that Dr. Blair could suppose that "the *structure* of the soul" was capable of alteration merely by the "opportunity being given for certain dispositions to exert themselves without restraint." Even if we admit that the passions and propensities came with the immortal soul into the material fabric, still we must strenuously maintain that *their* manifestations, like the manifestations of the soul itself, are entirely through the medium of corporeal organs—which is all that the phrenologists contend for. But while we make this admission of the fundamental principle of phrenology, and that upon the most deliberate reflection, we are not at all convinced that the phrenologists have proceeded with sufficient caution or prudence in their localisation of the organs through which these passions and propensities are manifested. If we are yet unable, after two thousand years of observation and reasoning, to ascertain the nature and seat of the most common corporeal diseases, is it likely that, in 24 years, we should be able to ascertain those portions of brain which are destined for the different faculties of the mind? With every friendly disposition to phrenology, we have grieved to see the precocious attempts at perfecting a system which we believe to be incapable of perfection. Nosology is surely subordinate to phrenology; and yet the greatest minds that the world has produced have utterly failed in giving it even the semblance of a science! Let not this discourage the phrenologist—let it incite him to industry—but let it teach him meekness—and, above all, let it render him distrustful of deductions hastily drawn in a science so new and so difficult as that of phrenology!

But, to the subject of this paper. The horrors of mind which have been created by the late atrocious murders in Edinburgh, must naturally inspire great curiosity as to the moral and physical characters of the criminals. We shall first lay before our readers a phrenological description of the cerebral organs of Burk. The measurements and developments are as follow:—omitting most of the organs unimportant as to the present subject.

MEASUREMENTS.

| | Inches. |
|---|---|
| "Spine to Individuality, . . . | 8 |
| Concentrativeness to Comparison | $7\frac{3}{8}$ |
| Ear to Occipital Spine, . . . | $4\frac{4}{8}$ |
| — to Individuality, . . . . | $5\frac{2}{8}$ |
| — to Firmness . . . . . . . | $5\frac{7}{8}$ |
| Destructiveness to Destructiveness, | $6\frac{3}{8}$ |
| Secretiveness to Secretiveness, . | $6\frac{2}{8}$ |
| Ideality to Ideality, . . . . | $4\frac{6}{8}$ |
| Constructiveness to Constructiveness, . . . . . . . . . | $5\frac{2}{8}$ |
| Cautiousness to Cautiousness, . | $5\frac{6}{8}$ |

DEVELOPMENT.

1. Amativeness, large.
2. Philoprogenitiveness, r. large.
3. Concentrativeness, rather full.
4. Adhesiveness, full.
5. Combativeness, full.
6. Destructiveness, very large.
7. Constructiveness, full.
8. Acquisitiveness, rather large.
9. Secretiveness, very large.
10. Self-esteem, very large.
11. Love of Approbation, full.
12. Cautiousness, large.

13. Benevolence, full.
14. Veneration, full.
15. Hope, on one side, small; on other, full.
16. Ideality, small.
17. Conscientionsness, full.
18. Firmness, large.
19. Individuality, full.

"Burk's head is rather above the middle size. The posterior lobe of the brain is large, and the middle lobe, in which are situate the organs of Destructiveness, Secretiveness, and Acquisitiveness, is very large; at Destructiveness in particular the skull presented a distinct swell. The anterior lobe, or that of intellect, although small in proportion to the hind and middle lobes, is still fairly developed, especially in the lower region connected with the perceptive faculties. Self-esteem is prominent, and has indented its form distinctly on the skull.

"The general result of this development is, that the animal feelings are very strong; the moral feelings are proportionately feebler, but not wanting; while observing intellect is present in a considerable degree, but reflecting intellect much less."

Thus, if the phrenologists are right in these admeasurements, and if they are capable of ascertaining the localities of the instruments of the passions, we see, as might be expected, a mixture of good and evil ones. Let us glance at the history of Burk, as diligently collected by Mr. Ireland, Junr.

This man was born in the North of Ireland, and was 36 years of age when he expiated his crimes on the scaffold. His father was a cotter, and gave young Burk a better education than is usually obtained by people in that rank of society. At school he was an apt scholar, cleanly, active, and good looking. He was taken into the service of a clergyman—afterwards into the service of a gentleman in Strabane—but ultimately he enlisted in the Donegal Militia. The regiment being disbanded, he came to Scotland, and worked on the Union canal, where he became associated with the woman Macdougall. Afterwards he came to Edinburgh, and lodged in one of those haunts of wretchedness and vice quaintly entitled, the "BEGGARS' HOTELS." The accidental death of a lodger in Hare's house, seems to have started the first idea of the horrible traffic which was afterwards carried on in human life. The body was sold to a Lecturer, and the money being expended, Burk decoyed a woman into the fatal lair, and for the first time stained his hands with blood! Conscience being once stifled, the sword was drawn against nature, and the scabbard was thrown away. Burk and Hare repeated similar tragedies sixteen times in one year! But though the feelings of humanity may be smothered, murder will not sleep—and detection ensues. The end of Burk is known. The wretched sequel of Hare's existence is too horrible to be imagined. Consumed by the fires of a hell within his own bosom, and spurned from the habitations of man, wherever his infamy is known, this loathsome stain on human nature, this melancholy blemish on the works of our common Creator, has, by adding treachery to murder, exchanged the momentary expiation of the scaffold, for years of unutterable remorse and indescribable torture!

And yet, the history of Burk, if correct, will shew that the direful passions or propensities of his nature lay dormant, as it were, till penury and opportunity opened the prospect of gain which the good dispositions of his mind were unable to resist, thus verifying the observations of Blair, which we have quoted above. The organs (phrenologically speaking) of destructiveness and benevolence counterbalanced or neutralised each other under ordinary circumstances; but the want of bread, the influence of bad company, and the temptation which restrictive laws on dissection held out, kicked the balance, and he plunged into guilt of the deepest dye.

It is no argument against the doctrines of phrenology that the organs of benevolence and conscientiousness, for example, should be found in such a man as Burk. The phrenologist admits the existence of the good as well as the bad organs, in all men—just as the divine (say Dr. Blair) assures us "the seeds of good and bad qualities lie in all our hearts." But surely it is more philosophical and infinitely more physiological to say, that the organs of our propensities lie in the head, than that the seeds of them lie in our hearts. If the antiphrenologists can give us any intelligible explanation of this latter theory, we are quite ready to abandon phrenology.

